# Passive Sensing of Preteens’ Smartphone Use: An Adolescent Brain Cognitive Development (ABCD) Cohort Substudy

**DOI:** 10.2196/29426

**Published:** 2021-10-18

**Authors:** Natasha E Wade, Joseph M Ortigara, Ryan M Sullivan, Rachel L Tomko, Florence J Breslin, Fiona C Baker, Bernard F Fuemmeler, Katia Delrahim Howlett, Krista M Lisdahl, Andrew T Marshall, Michael J Mason, Michael C Neale, Lindsay M Squeglia, Dana L Wolff-Hughes, Susan F Tapert, Kara S Bagot, ABCD Novel Technologies Workgroup

**Affiliations:** 1 University of California, San Diego La Jolla, CA United States; 2 University of Vermont Burlington, VT United States; 3 University of Wisconsin-Milwaukee Milwaukee, WI United States; 4 Medical University of South Carolina Charleston, SC United States; 5 Laureate Institute for Brain Research Tulsa, OK United States; 6 SRI International Menlo Park, CA United States; 7 Virginia Commonwealth University Richmond, VA United States; 8 National Institutes of Health Bethesda, MD United States; 9 Children's Hospital Los Angeles Los Angeles, CA United States; 10 University of Tennessee Knoxville, TN United States; 11 Icahn School of Medicine at Mount Sinai New York, NY United States; 12 See Acknowledgments

**Keywords:** preadolescents, smartphone use, passive sensing, screen use, screen time, mobile phone

## Abstract

**Background:**

Concerns abound regarding childhood smartphone use, but studies to date have largely relied on self-reported screen use. Self-reporting of screen use is known to be misreported by pediatric samples and their parents, limiting the accurate determination of the impact of screen use on social, emotional, and cognitive development. Thus, a more passive, objective measurement of smartphone screen use among children is needed.

**Objective:**

This study aims to passively sense smartphone screen use by time and types of apps used in a pilot sample of children and to assess the feasibility of passive sensing in a larger longitudinal sample.

**Methods:**

The Adolescent Brain Cognitive Development (ABCD) study used passive, objective phone app methods for assessing smartphone screen use over 4 weeks in 2019-2020 in a subsample of 67 participants (aged 11-12 years; 31/67, 46% female; 23/67, 34% White). Children and their parents both reported average smartphone screen use before and after the study period, and they completed a questionnaire regarding the acceptability of the study protocol. Descriptive statistics for smartphone screen use, app use, and protocol feasibility and acceptability were reviewed. Analyses of variance were run to assess differences in categorical app use by demographics. Self-report and parent report were correlated with passive sensing data.

**Results:**

Self-report of smartphone screen use was partly consistent with objective measurement (*r*=0.49), although objective data indicated that children used their phones more than they reported. Passive sensing revealed the most common types of apps used were for streaming (mean 1 hour 57 minutes per day, SD 1 hour 32 minutes), communication (mean 48 minutes per day, SD 1 hour 17 minutes), gaming (mean 41 minutes per day, SD 41 minutes), and social media (mean 36 minutes per day, SD 1 hour 7 minutes). Passive sensing of smartphone screen use was generally acceptable to children (43/62, 69%) and parents (53/62, 85%).

**Conclusions:**

The results of passive, objective sensing suggest that children use their phones more than they self-report. Therefore, use of more robust methods for objective data collection is necessary and feasible in pediatric samples. These data may then more accurately reflect the impact of smartphone screen use on behavioral and emotional functioning. Accordingly, the ABCD study is implementing a passive sensing protocol in the full ABCD cohort. Taken together, passive assessment with a phone app provided objective, low-burden, novel, informative data about preteen smartphone screen use.

## Introduction

### Background

Considerable neuromaturation [1,2] and cognitive development [1,3] occur during childhood and adolescence. Screen use, including a myriad of behaviors displayed using or in front of a digital device, may relate to cognitive and neurodevelopmental outcomes even in young children and preadolescents, especially among those who report high use (5 or more hours per day of screen use; [4,5]). For example, previous research suggests that childhood screen use may be associated with both positive and negative neurodevelopmental correlates, depending on individual differences [6]. Studies in older adolescents and adults indicate that smartphone screen use may be associated with deficits in attention, memory, reward processing, and overall functioning, although the extant literature is limited and conflicting [7]. For instance, general technology use in adults has a positive relationship with brain health, such as higher visual attention performance in video game players or greater use of the internet being related to more complex thinking abilities [8], although there is a dearth of studies on positive associations between screen use and brain health in children. Accurate assessment of brain-behavior relationships with screen use requires the accurate measurement of screen use, which is currently understudied.

One challenge to understanding the potential influence of screen use on brain development is that the measurement of screen use has typically relied on self-report [9-11] and parental report of the child’s screen use [12]. Self-reported screen use reports tend to underestimate actual use; for example, self-report data suggested that adults were using their smartphone for an average of 4.12 hours per day, whereas objective measurement indicated that the actual number was closer to 5.05 hours per day [9]. A recent meta-analysis of adults also found that fewer than 10% of self-reported screen use times by participants were within 5% of objective measurements [13]. It has been argued that current concerns about youth smartphone use and its consequences cannot be validated due to errors in self-report [13]. However, precise measurement may improve estimates of the impact of both quantity and type of smartphone-based screen use on psychological functioning and brain development in youth [14]. As such, new methods of measurement are needed to directly examine the quantity and quality of smartphone screen use to capture screen time–related behaviors (as in, the ways in which individuals use their screens) and potential associated screen-related pathology [15-17]. To date, data from objective assessment methods have been primarily limited to adults and older adolescents [16,17], parents of children [18], and young children [19], despite many children owning smartphones beginning in midchildhood [20,21]. Notably, a recent study in older children (aged 10-14 years) found passive monitoring combined with ecological momentary assessment (EMA) notifications to be feasible and acceptable [22]. However, the app used by Domoff et al [22] did not calculate exact app use, leaving a gap in the literature regarding which types of apps are most commonly used among children and adolescents.

Passive sensing via smartphones is promising in its potential to unobtrusively collect objective data on screen use [15]. Admittedly, even passive monitoring may elicit some form of demand characteristics, Hawthorne-type effect, or a novel way of thinking about queried items that then elicits new or different responses [23]. Some studies have theorized that knowledge of tracking of activity on a mobile device alone likely influences the behavior of research participants [24]. Consistent with this, studies using accelerometer data for physical activity [25] and smartphone use in youth [22] show differences in participant engagement during monitoring, although the real-world significance of these changes may be minimal [22]. Furthermore, a recent meta-analysis suggested that these methods can still more accurately inform correlates of screen use, purportedly doing so more accurately than participant report alone [13], making them a valuable contribution to scientific methodology.

The Adolescent Brain Cognitive Development (ABCD) study is a landmark longitudinal study of nearly 12,000 children aged 9-10 years who are being followed for at least 10 years. The design of the ABCD study investigates the impact of environmental exposures (such as screen use) throughout development on behavior and brain structure and functioning. Since its inception, this study has implemented various novel technological subjective and objective methods to assess and track behaviors [26]. An early target was the use of a passive monitoring smartphone app to assess smartphone screen use [26].

Acquisition of high-quality smartphone screen use data in children is a significant contribution to the field. Although research has demonstrated increasing screen use in children and adolescents in recent years [4], less is known about smartphone screen use specifics, such as when smartphones are used most, which apps or platforms are used, and for how long. Existing objective smartphone data are limited to largely adult and college-aged samples [16] and occasional preschool populations [27], with a recent addition of one study of older children and young adolescents [22]. However, a recent systematic review found that only 3 studies had investigated specific app use [16]. As the level of engagement with specific types of apps may be an important influence on mental health and other outcomes [28], research is greatly needed to refine passive (without direct participant engagement) and objective (rather than perceived) smartphone assessment approaches in children.

### Objectives

Accordingly, a pilot substudy within the ABCD study was designed to passively capture objective smartphone screen use data from preteen ABCD participants. These data were used to inform the acceptability and feasibility of the implementation of passive, objective assessment on a large scale across the ABCD cohort. In this study, we describe the development of an ABCD study passive monitoring app downloaded to participants’ phones, descriptive results from both self- and parent reports, and passive sensing of smartphone screen use among children. Beyond the novel descriptive information of children’s smartphone screen use, the primary aim is to assess the correspondence between self-report and passive sensing. We hypothesized that (1) child participants would underreport the amount of time they spent on their smartphones, relative to passive, objective measurement, and (2) the degree of child underreporting smartphone use would diminish with participation in a substudy focused on device use, with higher levels of self-reported screen use in the post- than in presensing periods. In addition, we aimed to assess the acceptability of passive sensing methods in a child population, expecting that children and parents would find passive sensing of the child’s smartphone to be acceptable.

## Methods

### Participants

Four ABCD study sites that were roughly geographically dispersed among all ABCD study sites participated in this pilot substudy project. Full details regarding the larger ABCD study design are provided elsewhere [29]. All ABCD study participants who were at a substudy site for their 2-year follow-up and had a study-compatible Android smartphone were invited to participate in the substudy between August 2019 and January 2020. Eligible ABCD study participants were invited to install the ABCD-specific Effortless Assessment of Risk States (EARS) [30] app on their phones for at least 4 weeks. Several weeks after the substudy launch, data were collected regarding the number of participants invited and reasons for declining substudy participation, if relevant. Of those invited, approximately 40% were ineligible due to having an iPhone, 31% did not have their own smartphone device, and 8% had other incompatible devices. Of those eligible with compatible devices, 4% of the invited sample (around 20% of those eligible with compatible devices) declined to enroll in the substudy. A total of 71 participant-parent dyads assented and consented to participate, respectively; 4 participants did not complete the full substudy protocol, and therefore, their data were not included in the present analyses. Thus, a total of 67 participants were enrolled in the study, and they downloaded the EARS app onto their phones. Participant-parent dyads provided written informed assent and consent. All study protocols were approved by the institutional review board.

### Measures

#### Demographics

The ABCD study collects full demographic data, including age, sex, parental income, race, and ethnicity [31]. In addition, participants were queried as to their self-identified gender; no participants identified as being transgender.

#### Screen Time Questions

Before participating in the substudy, participants and their parents independently completed a questionnaire specific to the child’s smartphone device use (presensing). The questionnaire included a 20-item screen time self-report measure modified from previous research [32,33] that assessed how much time participants spent on their phone over the past 4 weeks (“How much of [the time on a weekday/weekend] do you/does your child spend on their mobile device specifically?”) and other health and behavior questions (eg, concerns about time spent on the phone). Weekday and weekend averages were reported for both overall time on media on their smartphone and specific types of media use on any device (ie, streaming television or movies, streaming videos, playing single-player or multi-player video games, texting, on social media, editing videos or pictures for social media, browsing the internet, and total time). At the end of the 4-week data collection period, participants and parents were asked the same questions about the child’s smartphone use and about times when they were without their phone during the 4 weeks they had the EARS app installed on their mobile device (postsensing). For both presensing and postsensing assessments, average daily self- and parent-reported smartphone use was calculated by adding weekday hours multiplied by 5 and weekend day hours multiplied by 2, then dividing by 7.

#### EARS App

Given prior research indicating concerns of battery life, acceptability, and privacy [34], and participant ages 11-12 years, a relatively brief window of 4 weeks of passive sensing data collection was selected. Although commercial or operating system–specific apps (eg, Digital Wellbeing, Apple Health) were considered [26], an app previously used in pediatric research and customized for ABCD was chosen for its optimal function and safety. A collaborative relationship was entered with Ksana Health, creator of the EARS app [30]. Ksana Health customized their passive sensing app for download onto ABCD participants’ smartphones for data collection. Owing to potential privacy concerns, this customized version of the EARS app for the ABCD study only collects smartphone data on the duration and time of day of specific apps’ use. Tools such as language capture and semantic categorization, geolocation, music or mood profiles, and EMA are available in EARS but were not incorporated into the current ABCD study version.

The ABCD study research assistants helped participants download the ABCD study version of the EARS app from the Google Play Store. On opening the EARS app, the research assistant scanned a code that linked the participant’s ABCD study unique participant number to their device and EARS app data. This approach allowed for a completely confidential conveyance of data and the participant’s identification remained confidential. The EARS app ran continuously in the background of the participant’s phone, scraping the operating system every few minutes to collect information on (1) screen on and off and (2) which app was in the foreground. Date and time were logged for each app use instance. In addition to individual app use information (data not presented here; accessible through [35]), Ksana Health reviewed each individual app used by a participant and computed summaries across composite app categories: communication (eg, Discord; Facebook), gaming (eg, Temple Run 2, Mario Kart Tour), music (eg, Shazam, Google Play Music), news (eg, Weather Forecast, HuffPost News), reading (eg, WebComics, Amazon Kindle), social media (eg, Twitter, Facebook, TikTok), and streaming (eg, Twitch, Hulu). Certain apps were listed in multiple categories because of the multiple ways in which they can be used (eg, Facebook in both communication and social media). This was done to make more comprehensive categories, although consequently no category is mutually exclusive or independent of one another. In addition, 2 stand-alone categories were created from popular apps: SMS messages (basic texting) and YouTube. Stand-alone categories were not mutually exclusive from composite categories (eg, YouTube was also counted within streaming). Although Google Play Store category summaries are available in ABCD Annual Release 3.0 through National Institute of Mental Health Data Archive (NDA), here we limited analyses to newly created categories that better fit types of apps of interest at this age (eg, social media, gaming). All collected data were encrypted before being uploaded to a secure cloud server. Neither identifiable information (such as participant’s name, age, or phone number) nor content regarding what participants were doing was collected. If a participant stopped the EARS app from running in the background, once function resumed, EARS then queried the operating system to collect overall screen use information. This allowed for collection of all screen use, although some finer details (eg, time of day) may not have always been obtained. Raw participant file data accessed via National Institute of Mental Health Data Archive [35] were reviewed to assess for any full days of missing data; no participants had a full-day gap in data collection, indicating no missing passively sensed data.

Participants were not asked to interact with the EARS app, and if they opened it, they would see the message, “You are changing the future of health and wellness.” If data were not being received from the phone, the app would push a notification to the participant to open the app, allowing for continuous data collection with minimal intervention. If a participant did not respond to notifications for several days, an ABCD staff member contacted the participant to ensure they still had their phone and the EARS app and troubleshooted, as necessary.

The EARS app for the pilot substudy was limited to use on Android phones with operating systems 6.0 or newer. Although Apple products were considered, the iOS operating system does not allow passive scraping (collecting) of app use information, precluding the inclusion of Apple smartphones from being included in this pilot. In the ABCD data release NDA 3.0, for the 6571 participants at year 2 follow-up (when the substudy occurred), 36.95% (2428/6571) of the participants had no smartphone, 37.79% (2483/6571) had an iPhone, 23.07% (1516/6571) had an Android, and 2.19% (144/6571) either had another type of smartphone or refused to answer.

### Statistical Analysis

Analyses were run in R 3.6.1 [36] using RStudio [37]. Summary descriptive statistics were calculated for total app use time, total app category time, and self- and parent-reported smartphone use. Analysis of variance evaluated potential differences in the most commonly used app categories (streaming, communication, and social media) by demographic factors (age, sex, household income, race and ethnicity, and geographic location). To assess self-report relative to passive, objective sensing data, Pearson correlations were run to assess relationships between passive, objective sensing daily smartphone use and postsensing self- or parent reports. Postsensing reports were used, as these better reflected the sensing period when objective data were collected. Significant correlations between objective and self-report measurements were then tested using paired sample two-sample *t* tests and chi-square tests. For our second aim, paired samples *t* tests were also used to test mean differences in self-report of pre- and postsensing and self- and parent-report. Finally, to assess the acceptability of passive, objective sensing data collection, the total percentage of child and parent participants’ willingness to have the app on the child’s phone for longer was reviewed. All *P* values <.05 were interpreted as significant.

## Results

### Overview

The substudy participants (36 men and 31 women; 23/67, 34% White; 5/67, 8% Black; 20/67, 30% Hispanic; 1/67, 2% Asian; and 18/67, 27% *Other*) were at their ABCD year 2 follow-up (mean age 11.88, SD 0.7 years; range 10.75-13.17 years). The annual household income was <US $50,000 for 35% (23/65), US $50,000-$99,999 per year for 34% (22/65), and >US $100,000 per year for 31% (20/65) of the participants. Comparison of demographics between the full baseline ABCD study cohort and the present sample is presented in Multimedia Appendix 1.

At presensing, 53% (35/66) of the children reported that their parents limit their screen use, with 42% (28/66) of the parent participants restricting smartphone use specifically. Overall, 32% (31/66) of the children reported that their parents installed device monitoring apps on their phone. Similarly, 32% (31/66) of the parents reported having a parental monitoring app on their child’s phone and 29% (19/66) reported restricting their child’s screen use every day, whereas 42% (28/66) reported rules for weekdays but not weekends, 15% (10/66) reported no rules, and 14% (9/66) said they had not yet made rules. Passive sensing data collection spanned an average of 33.91 (SD 22.20) days to collect the requisite 4 weeks of data, of which 24.22 were weekdays and 9.69 were weekend days, on average. Participants used an average of 2.25 (SD 1.25) unique apps per day, with a total average of 62.48 (SD 22.45) unique apps over the course of sensing. Specific app use is listed in Table 1.

**Table 1:** Average app use (total time) sensed by Effortless Assessment of Risk States from child’s smartphone during the 4-week passive sensing period (N=67)^a^.

Category	Use time, mean (SD)
Streaming	1 h 57 min (1 h 32 min)
YouTube	1 h 18 min (1 h 23 min)
Communication	48 min (1 h 17 min)
Gaming	41 min (41 min)
Social media	36 min (1 h 7 min)
SMS messages	6 min (11 min)
Reading	3 min (10 min)
Music^b^	2 min (5 min)
News	1 min (2 min)

^a^Categories are composites of specific apps, with the exception of SMS messages and YouTube. Multifunctional apps were listed in multiple categories (eg, Facebook in both communication and social media), so no category is mutually exclusive. SMS messages and YouTube were not mutually exclusive from composite categories (eg, YouTube was also counted within streaming).

^b^Music frequently runs in the background and is only in the foreground when actively selecting songs or turning the app on and off (measured here).

### Relationship Between Objective and Self-report Data

**Figure 1:**
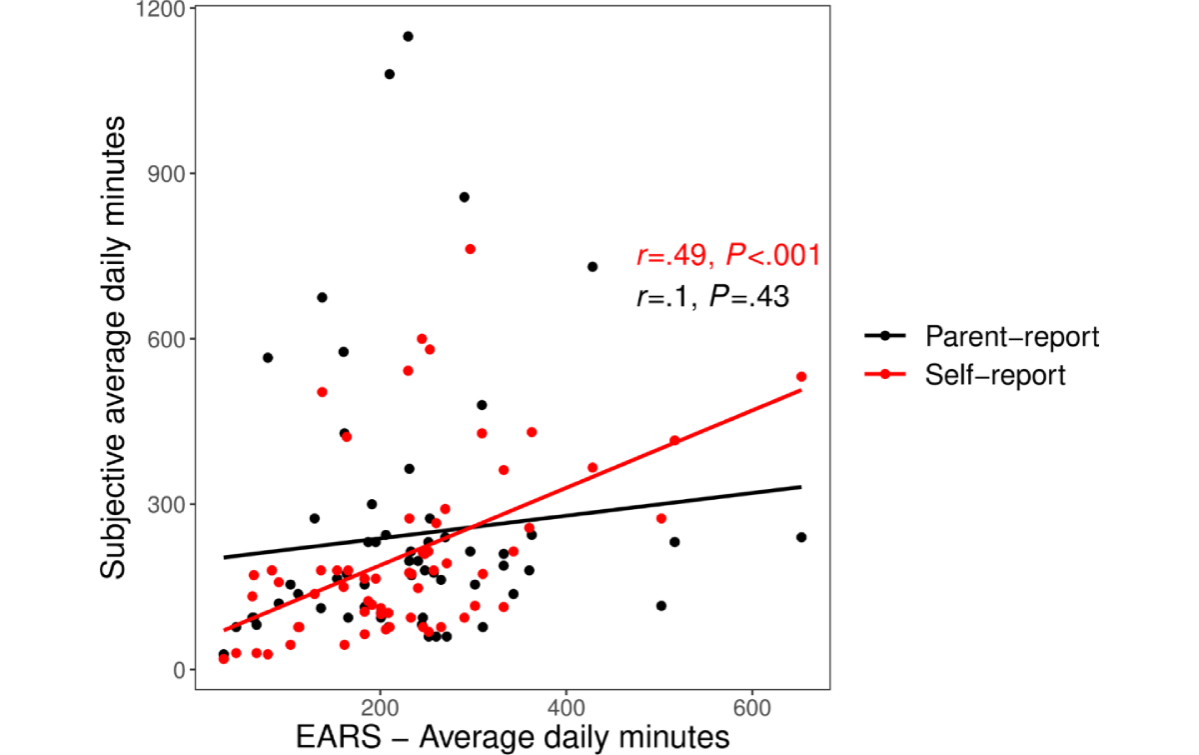
Scatterplot of average daily smartphone use by Effortless Assessment of Risk States and postsensing self-report (in red) and postsensing parent-report (in black). Best-fit simple regression lines are represented for parent report (black) and self-report (red). EARS: Effortless Assessment of Risk States.

Responses for self- and parent-reports of average daily smartphone screen use before and during the sensing period are reported in Table 2, along with the average daily use as measured by the EARS app. Passive, objectively sensed, and self-reported daily smartphone screen use were significantly correlated (*P*<.001; *r*=0.49; 95% CI 0.28-0.66; Figure 1). Retrospective self-report for the weeks of the sensing did not significantly differ from passive, objective data (*P*=.32), with a nonsignificant tendency for children to show lower and more variable (ie, wider range) self-report than passively collected objective data. However, self-report data collected at presensing about use over the 4 weeks before the sensing period were significantly less than passive, objective data (*P*<.001). Overall, 79% (53/66) of children reported less smartphone screen use than indicated by passive sensing, and 21% (14/66) reported more smartphone screen use (Χ^2^_1_=21.8; *P*<.001). Parent report of their child’s smartphone screen use at both pre- (*r*=0.22) and postsensing (*r*=−0.06) did not significantly correlate with objective measurement or with children’s self-report (*P*>.05). In the postsensing report, 58% (39/67) of the parents reported less screen use than indicated by passive, objective sensing, and 42% (28/67) reported more (Χ^2^_1_=1.6; *P*=.20). As parent report varied widely, with some parents reporting their child using their smartphone for more than half the day, results were reanalyzed excluding reports that were 3 SD above the mean; results remained unchanged.

**Table 2:** Child- and parent-report compared with Effortless Assessment of Risk States (EARS) app data for total average daily smartphone screen use, before and after the 4-week passive sensing period (N=67).

	Daily device use (4 weeks before sensing), mean (SD)	Daily device use (during 4 weeks of sensing), mean (SD)
Self-report	2 h 45 min (2 h 31 min)	3 h 28 min (2 h 43 min)
Parent report	4 h 3 min (3 h 3 min)	4 h 4 min (3 h 51 min)
EARS app use	N/A^a^	3 h 45 min (1 h 55 min)

^a^N/A: not applicable.

### Pre-and Postsensing Behavior Changes

Child self-report of daily average device use pre- and postsensing were correlated (*r*=0.41; *P*<.001; 95% CI 0.19-0.60) and showed an increase (Table 2) from the pre- to postsensing period (*t*_1,65_=−2.48; *P*=.02; 95% CI −93.97 to −10.06). Parents’ pre- and postsensing reports of their child’s smartphone screen use were also correlated (*r*=0.61; *P*<.001; 95% CI 0.43-0.74), with no difference between their pre- and postsensing reports (*P*=.63). Most participants reported changing their smartphone screen use behavior during the 4-week sensing period *a lot* (33/62, 55%) or *a little* (28/62, 42%), with only 3% (1/62) reporting *not at all*. Most parents reported no change in monitoring their child’s smartphone screen use (42/62, 67%), whereas 25% (15/62) reported closer monitoring and 8% (5/62) did not know; only 8% (5/62) reported a change in their child’s smartphone screen use (which was typically an increase). Before sensing, 21% (14/66) of the children reported using the phone more than others their age, which reduced to 13% (8/62) at the postsensing assessment, and children with higher passive, objective screen use tended to perceive that they used their smartphone more than their peers before (*r*=0.33) and after (*r*=0.54) passive sensing.

### Feasibility and Acceptability of Passive Assessment of Smartphone Screen Use

Children reported not accessing their phones for a mean of 4.94 (SD 5.04) cumulative days, and parents reported that their child did not use their phone for 4.42 (SD 5.15) days during the 4-week sensing period; however, even in instances when the participant may not have actively used their phone, the EARS app still scanned the device for screen use. Most participants (43/62, 69%) and their parents (53/62, 85%) reported willingness to have a monitoring app such as EARS on the child’s phone for a longer period, with 18% (11/62) of child participants unsure.

### Demographic Differences in Smartphone Screen Use

**Figure 2:**
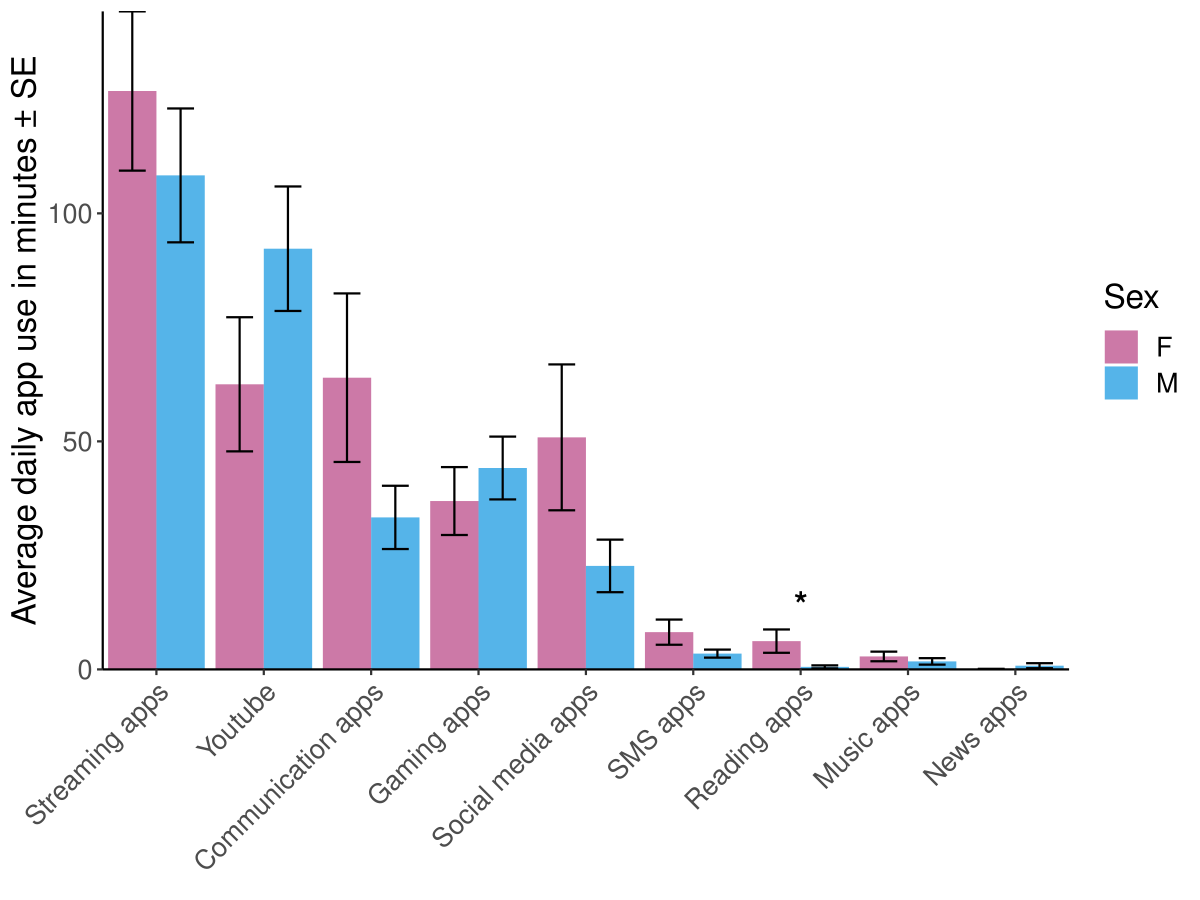
Average daily app category use sensed by Effortless Assessment of Risk States for girls and boys in minutes. The only significant difference between girls and boys was in reading apps (F_1,65_=5.55; *P*=.02). All other app comparisons were not significant at *P*>.05. F: female; M: male.

Girls showed more average daily passive, objectively sensed smartphone screen use than boys (4 hours 17 minutes vs 3 hours 19 minutes, respectively; *F*_1,65_=4.48; *P*=.04) and showed a higher average daily use of reading apps (6 minutes vs <1 minute, respectively; *F*_1,65_=5.55; *P*=.02; Figure 2). No other demographic differences (age, sex, household income, race and ethnicity, or geographic location) were observed for passive, objectively sensed use of the smartphone overall or for app types. There were also no differences according to demographics for postsensing self-report (sex, *P*=.16; age, *P*=.35; race and ethnicity, *P*=.82; income, *P*=.48; geographic location, *P*=.35) or parent-report (sex, *P*=.51; age, *P*=.92; race and ethnicity, *P*=.72; income, *P*=.06; geographic location, *P*=.14).

## Discussion

### Principal Findings

Longitudinal objective smartphone screen use data from a large cohort of children are needed to assess trajectories of smartphone screen use with regard to time spent in total and on specific apps, as well as to map changes in types of information accessed and behaviors displayed across platforms. These data can further be linked to changes in behavior, brain development, psychopathology, and health outcomes across development. In this study, novel data suggests that this type of information is feasible to collect within the ABCD cohort and reveals important information regarding how children are using their smartphones.

Descriptive data from these analyses indicate a wide variety of app use in children. Novel passively sensed data suggest streaming as the most common use of smartphone devices. As defined within the composite category, streaming includes apps such as Netflix and Hulu, as well as others such as Twitch, TikTok, and YouTube. Although streaming is often thought of as a more passive activity, the inclusion of apps with social properties (such as TikTok) also allows for more active engagement. The next most common app categories were communication, gaming, and social media. Thus, it appears that a combination of entertainment and social connection are the primary drivers of smartphone screen use, as most included apps have the dual capability of passive viewing and active socializing (eg, commenting, posting videos). Previous reports of adolescent and young adult screen use, and particularly social media use, indicate that teens are motivated by social needs [38,39], entertainment [39,40], communication with peers and group members [41-43], complying with perceived social norms [44], feeling a sense of belonging [41], and agency and identity formation [45]. Social motivators are particularly important in adolescence, as teens are likely to seek social approval even at risk of other harm or negative outcomes [46]. The current use patterns may then map well onto reasons for use, as suggested by the literature, despite the young age range of the present sample. In addition to motivations for use, types of screen use and levels of engagement have previously been suggested to be important in screen use outcomes [47,48] and warrant further research.

Perhaps unsurprisingly given their age, other app categories such as *News* were minimally used by this sample. Children may also rely on nontraditional sources of news, such as social media. In addition, although it appears that *Music* apps were used at similarly low rates, this is likely a limitation of the measurement methodology. The EARS app measures apps in the foreground; however, many music-based apps are now able to continue playing music in the background once an album, artist, or playlist are selected. Thus, the *Music* category is likely an underestimate of actual listening time and *Music* in general appears to be a more passive form of engagement, as children are not frequently actively selecting songs or artists. Together, the wide range of category-based data is informative for the basic description of preadolescent children’s smartphone habits, although it also raises many more questions, such as the influence of background apps that require more sophisticated analyses and methods to elucidate.

One of the primary findings of this study is that, assuming relative stability in amount of screen use over time, children underreported smartphone use on presensing, with a stronger correlation (though still moderate in strength) between perceived and measured screen use after the month-long monitoring period. Notably, however, without data from the presensing period, we cannot confirm that participants underreported use rather than actually used their smartphones less. Furthermore, parental report of their child’s smartphone screen use was not significantly correlated with passive, objective measurement of use, or with self-reported data. Consistent with concerns from previous research [9-12,49], these findings suggest that reliance on self- or parent-reported estimates of smartphone screen use is likely inaccurately assessing the effects of screens and smartphone use in children and adolescents. Notably though, children appear to be more accurate reporters than their parents. Thus, the collection of passive, objective data of smartphone screen use, rather than reliance on either parent or self-report alone, may contribute more accurate and informative data for understanding how smartphone screen use shapes brain-behavior and pathology relationships in children.

Descriptive data from parent report indicate that parents generally overreported their child’s average daily smartphone screen use when looking at the overall mean use reported. On balance, 58% (39/67) of parents underreported their child’s smartphone screen use when comparing parent report with passive, objectively sensed data. Similarly, one study found that parents may overreport, relative to their children, screen use [50], although others have found underreporting by parents that they suspect was due to a social desirability bias [49]. As found here, parents are likely unaware of the extent and ways their preteen children use smartphones, as manifested here through inaccurate reporting that is both above and below actual screen use. Together, this suggests decreased ability to adequately monitor their child’s behaviors. This is concerning as decreased parental monitoring has been implicated in increased problematic behavior in adolescents [51,52].

Interestingly, children’s self-report became more consistent with the objective data after completing the passive sensing protocol (postsensing) and a majority endorsed their phone use behaviors changed *a lot* during the study period. This finding may indicate that, despite the passivity of app-based smartphone measurement, any type of monitoring could influence behavior. It also may be that perceived screen use may fall in line more with passively collected metrics given that participants’ attention was drawn to their screen use after being queried about it. A study of passively collected physical data using an accelerometer similarly found participant-reactivity to monitoring [25]. In another study of children and young adolescents, Domoff et al [22] found passive sensing of smartphone use to statistically, but not meaningfully (approximately 11 minutes per day), increased smartphone screen time. Other research indicates that knowledge of being observed alone can change behavior [23]. Although ABCD and this substudy were designed to be purely observational, the pilot nature of app development may have required additional intervention from the study team (ie, app notifications; calls from research assistants to query whether the app was still installed on the phone). Even if data collection is modestly influencing behavior, the combination of objective and postsensing self-report data is likely more accurate than reliance on presensing self-report alone, as has been supported by a meta-analysis [13]. In addition, although moderately correlated, there is still great variability in self-report in relation to objective report. Although retrospective self-report may be less accurate, it is also possible that app use measurement may not always reflect actual use, as with music apps, and better app measurement is desirable. A combination of passive sensing and real-time self-report, such as through EMA, may provide more accurate and robust app use data.

Notably, the vast majority of children and their parents reported openness to extending the amount of time the EARS app was on their phone, indicating that the use of passive assessment of smartphone screen use is highly feasible and acceptable even in children. This is consistent with previous research on passive mobile sensing in parents and young children (aged 3-5 years) [18,19] and in similarly aged youth [22]. The types of data collected through the EARS app meet most of the recent recommendations for passive sensing smartphone research [16], including collecting data on general use time, screen on and off time, most used apps, and length of app use. Furthermore, raw data can be accessed through the National Institute of Mental Health Data Archive, allowing for the investigation of specific apps or creation of new researcher-derived composite categories outside of those provided by ABCD and Google Play Store categories. New composite categories created by individual investigators or research groups may be more informative than the included categories that are not mutually exclusive included apps. Summary data for daily, weekday, and weekend use are already provided within the NDA 3.0 data release, and combining composites with raw data could be used to further break down use patterns into the time of day that an app was used. Thus, the level of detail available within this subsample and, soon, the full ABCD cohort is uniquely rich and valuable for investigating smartphone screen use. On balance, the depth of available data suggests that further innovation is needed to process the amount of data, and best practices for using such fine-grained data should be outlined.

### Limitations

The data presented in this study are from a small subsection (62/11,875, 0.52%) of the overall ABCD participants and only includes Android users, significantly reducing generalizability from the full ABCD cohort. The ABCD-specific EARS app sensing design is only compatible with non-iOS devices because of Apple blocking app scraping programs, although additional components of EARS (eg, EMA) that are not used by ABCD can be implemented in iOS. Future ABCD passive sensing data collection with the EARS app include methods to calculate a proxy variable for time on Apple phones, which can be validated in comparison to Android data in future ABCD time points. App categories were created by adults, which may not directly translate to how children and adolescents actually use apps. Categories on their own may not be accurate. For example, although YouTube is generally viewed as a streaming app, it also has means for communicating with others, suggesting it may further be categorized as a communication or social media app. In addition, the youth only reported their overall smartphone screen use time, preventing assessment of the correlation between self-reported specific categories of app use (eg, social media apps) with objective measurement of that same category. Beginning in ABCD follow-up year 4, youth self-report categories of smartphone screen use so that this can be assessed. The methods used here only consider smartphone screen use, not capturing screen time from other platforms (eg, TV, tablets, computers, gaming systems). Some information can also not be derived, such as the frequency of download and removal rate of apps, despite the large range of unique apps used by children. As discussed above, passive monitoring may not have been as passive as desired because of phone notifications and research assistant troubleshooting contact. For the year 4 follow-up in the full cohort, processes are being further automated to attempt to reduce any potential intervention from reminders regarding app monitoring. In addition, participants reported perceived changes in smartphone screen use and greater time spent on their phone, and additional research is needed to determine whether their perceived increase in use was accurate and, if so, how and why they changed their use. App data use available in NDA 3.0 is rich and detailed, providing many potential avenues of investigation; given the nascent area of research, we limited our analyses to general overall findings. Future research should take a more fine-grained approach to investigate questions such as patterns of use (eg, do children use the same apps and app categories day after day; time of use). Finally, although the EARS app generally uses minimal battery [30], particularly in the ABCD version with fewer sensors activated, participants may still have noticed and been bothered by the battery or data drain, potentially further changing behavior.

### Conclusions

In summary, using passive, objective sensing of app use revealed novel information regarding the types and duration of app use by children. The monitoring system was generally viewed favorably by participants and their parents, and child-parent dyads reported willingness to continue to have the limited electronic passive sensing system on their personal smartphone for research purposes. The results suggested that child report was consistent with objective measurement, though only at a moderate level and with greater variability. Given the sparsity of high-quality data on smartphone use in this developing age group, more objective, longitudinal research in this domain would be beneficial for delineating how amount of time and types of engagement with smartphones impact physical, neurological, and mental health development, as will be assessed in the ABCD study.

## Appendix

Multimedia Appendix 1 Demographics for both the full Adolescent Brain Cognitive Development study cohort at baseline and the present subsample of participants at Year 2.

## Abbreviations

ABCD: Adolescent Brain Cognitive Development
EARS: Effortless Assessment of Risk States
EMA: ecological momentary assessment
NDA: National Institute of Mental Health Data Archive
