# Supplementary material for: Passive Sensing of Preteens’ Smartphone Use: An Adolescent Brain Cognitive Development (ABCD) Cohort Substudy
Source: JMIR Ment Health. 2021 Oct 18;8(10):e29426. doi: 10.2196/29426 (PMC8561413; doi:10.2196/29426)
Supplement: Multimedia Appendix 1 [file mental_v8i10e29426_app1.docx]

Supplemental Table 1. Demographics for both the full ABCD Study cohort at baseline and the present subsample of participants at Year 2.

|  |  | Full ABCD Study Cohort (Baseline)  N=11,878 | Pilot Study  Subsample  N=67 |
| --- | --- | --- | --- |
| % Female |  | 48% | 46% |
| Race/Ethnicity | White  Hispanic  Black  Asian  Other | 41%  21%  16%  2%  17% | 34%  30%  8%  2%  27% |
| Annual Household Income | <$50,000  ≥$50,000 & <$100,000  ≥$100,000 | 30%  28%  42% | 35%  34%  31% |
